# Two-Step Size-Exclusion Nanofiltration of Prothrombin Complex Concentrate Using Nanocellulose-Based Filter Paper

**DOI:** 10.3390/biomedicines8040069

**Published:** 2020-03-26

**Authors:** Levon Manukyan, Athanasios Mantas, Mikhail Razumikhin, Andrey Katalevsky, Eugen Golubev, Albert Mihranyan

**Affiliations:** 1 Nanotechnology and Functional Materials, Department of Materials Science and Engineering, Uppsala University, Box 534, 751 21 Uppsala, Sweden; levon.manukyan@angstrom.uu.se (L.M.); athanasios.mantas@angstrom.uu.se (A.M.); 2 Nacimbio JC, 10, 2nd Volkonsky lane, 127473 Moscow, Russia; razumikhin.m@gmail.com; 3 Biopharmgarant LLC, Stancionnaya 45, 600901 Vladimir, Russia; andreyalexandr2003@yandex.ru; 4 National Research Center for Hematology, Novyi Zykovskiy proezd 4, 125167 Moscow, Russia; ev-genius@bk.ru

**Keywords:** Mille-feuille filter, Cladophora cellulose, hemophilia B, protein aggregates, virus removal filtration

## Abstract

Coagulation Factor IX-rich protrhombin complex concentrate (FIX-PCC) is a therapeutic biologic product that consists of a mixture of several human plasma-derived proteins, useful for treating hemophilia B. Due to its complex composition, FIX-PCC is very challenging to bioprocess through virus removing nanofilters in order to ensure its biosafety. This article describes a two-step filtration process of FIX-PCC using a nanocellulose-based filter paper with tailored porosity. The filters were characterized with scanning electron microscopy (SEM), cryoporometry with differential scanning calorimetry, and nitrogen gas sorption. Furthermore, in order to probe the filter’s cut-off size rejection threshold, removal of small- and large-size model viruses, i.e., ΦX174 (28 nm) and PR772 (70 nm), was evaluated. The feed, pre-filtrate, and permeate solutions were characterized with mass-spectrometric proteomic analysis, dynamic light scattering (DLS), sodium dodecyl sulfate-polyacrylamide gel electrophoresis (SDS-PAGE), and analytical size-exclusion high-performance liquid chromatography (SEHPLC). By sequential filtration through 11 μm pre-filter and 33 μm virus removal filter paper, it was possible to achieve high product throughput and high virus removal capacity. The presented approach could potentially be applied for bioprocessing other protein-based drugs.

## 1. Introduction

Replacement therapy using plasma-derived Factor IX (FIX) products is a life-saving treatment for patients with hemophilia B. Both recombinant and plasma-derived FIX show high efficacy in clinical trials [1]. Production of FIX normally involves multiple steps. High purity FIX is obtained from prothrombin complex concentrate (PCC), which is a mixture of vitamin K-dependent clotting factors, e.g., factor II (prothrombin), V, VII, IX, and X, and clotting inhibitors, e.g., protein C, Z, and S [2]. PCC preparation is a highly complex mixture of proteins and may contain up to 50% of proteins other than FIX [3]. Both highly purified FIX and PCC can be used for hemophilia B treatment [4]. Also, PCC preparation may be useful for prevention of bleeding due to overdose of oral anticoagulants and liver dysfunctions [2,3].

As it is with all plasma-derived products, the viral safety of FIX-rich PCC is a critical issue [5]. According to current regulations, at least two orthogonal virus clearance steps must be implemented to ensure viral safety of the final product [6]. The steps to mitigate virus contamination of FIX and PCC products include donor screening for known blood-borne viruses, i.e., HIV 1–2, HBV, HCV, HAV, and parvo B19; virus inactivation, such as solvent/detergent, mixed chemical inactivation (tri-n-butyl phosphate) and detergent (nonionic, polysorbate, and polyethylene oxide) treatment; and incubating intermediate product in controlled temperature (usually 6 h at 25 °C) [3,7,8]. Dry heat treatment may also be used after lyophilization, e.g., 100 °C for 1 h or 80 °C for 72 h. In the past, steam treatment at 60 °C (190 mbar) for 10 h or 80 °C (375 mbar) for 1 h was reported [3,7,8]. Presently, virus removal nanofiltration has become widely used as a robust and reliable method for ensuring viral safety. Nanofiltration is attractive because it is capable of physically removing all types of viruses from protein solution as opposed to virus inactivation. Several authors have described the application of virus removal nanofiltration for FIX industrial products using Planova 15/20/35N [9,10,11,12,13,14,15], Viresolve NFP [11,16,17,18,19], and Ultipor DV50 filters [2]. The conclusion of these studies is that filtration of plasma-derived FIX-rich products is challenging due to the presence of large molecular weight impurities and protein aggregates.

It is known that the levels of different impurities are highly dependent on the type of chromatographic separation that was used during plasma treatment [3,18,20]. The most extensively described impurities in vitamin K-dependent clotting factors include inter-α-trypsin-inhibitor (ITI), complement 4b binding protein (C4BP), and vitronectin (VN). C4BP is a large glycoprotein of 570 kDa [21] in the shape of an octopus that consists of seven α-chains connected to a single β-chain by disulfide bonds [22,23,24]. The α-chains are responsible for binding C4b, while the β-chain has high-affinity for VN (protein S), forming large complexes [22,25]. The molecular conformation of C4BP is highly dependent on the surrounding medium composition [11,19]. In the charged state the α-chains repel each other, thereby occupying much larger volume than the same molecule in the uncharged state. In its open structure form, C4BP would have a diameter of approximately 66 nm since each α-chain arm is 33 nm long [11,22]. Varying salt concentrations have been shown to affect the compactness of C4BP molecule and thereby the flux properties of the virus removal filter [19]. Another large Mw impurity present often in FIX products is ITI. ITI (225 kDa) is a large complex that consists of one light and several heavy chains (H1-H3) covalently linked by a chondroitin sulfate chain. The heavy chains of ITI proteins function as hyaluronic acid (HA) binding proteins, whereas the light chain, also called bikunin, functions as a serine protease inhibitor upon activation [26]. The third extensively described impurity is VN [27]. The monomeric form of VN has a cryptic hydrophobic pocket, which upon exposure and conformational changes exhibits heparin- and C5b-7 binding activity [28]. Normally, only 2% of VN in plasma shows heparin-binding activity but its fraction increases manifold during coagulation [29]. VN also presents a free thiol group capable of disulfide bonding [30]. When unfolded VN is highly prone to polymerization and may form aggregates with Mw up to 1000 kDa [28]. Studies on nanofiltration of FIX products where VN aggregates were detectable confirmed its role as a filter foulant [27]. Extensive coverage of various impurities at different intermediate stages during FIX manufacturing is discussed elsewhere [15,18].

So far successful virus removal filtration of FIX products has been described in the literature only for a limited number of commercial filters. A novel type of virus removal filter paper was developed at Uppsala University, which is produced by adapting traditional paper making technology and consists of 100% cellulose nanofibers [31,32]. The pore size and flux properties of the filter paper can be controlled, which opens new opportunities to model fundamental aspects of bioprocessing [32,33,34]. The filter paper was previously validated in numerous studies to remove several large and small-size model viruses, including retroviruses (xMuLV, 100 nm) [35], parvoviruses (MVM, 20 nm) [32,36], and model phages (ΦX174, 28 nm) [37,38,39]. Recently, it was shown that this nanocellulose-based virus removal filter paper is useful for bioprocessing human plasma-derived IgG [40]. 

In this article, for the first time the filtration of FIX-rich PCC using a nanocellulose-based virus removal filter paper is described. Furthermore, a two-step size-exclusion nanofiltration process is developed to remove foulants and ensure efficient virus removal filtration of FIX-rich PCC using nanocellulose-based virus removal filter paper. FIX-rich PCC was used as a model for a highly complex plasma-derived product to simulate industrial bioprocesses where impurities may greatly affect product yield and biosafety.

## 2. Materials and Methods 

### 2.1. Materials 

Cladophora cellulose was provided by FMC Biopolymer (batch 3095-10; Newark, DE, USA). FIX-rich PCC was provided by National Center for Hematology, Moscow, Russia, as lyophilized powder. Coliphages ΦX174 (ATCC 13706-B1™) and PR772 (BAA-769-B1), and the host bacteria Escherichia coli (Migula) Castellani and Chalmers C (ATCC 13706) and K12 J53-1(R15) [HER 1221] (BAA-769) strains were obtained from ATCC (Manassas, VA, USA). Agar (214530) was obtained from BD (Franklin Lakes, NJ, USA). Tryptone (LP0042B) and yeast extract (Oxoid) (LP0021) were obtained from Thermo Fisher Scientific. Phosphate-buffered saline (P4417), 2-mercaptoethanol (M3148), sodium chloride (S5886), sodium phosphate dibasic (71640) and 2-mercaptoethanol (M3148) were purchased from Sigma-Aldrich (Saint Louis, MO, USA). Any kD™ Mini-PROTEAN^®^ TGX Stain-Free™ protein gels (4568125), tris/glycine/SDS running buffer (1610732), 4x Laemmli Sample Buffer (1610747), and Precision Plus Protein™ unstained protein standards (1610363) were purchased from Bio-Rad (Hercules, CA, USA).

### 2.2. Filter Preparation

Filters of different thickness were prepared from Cladophora cellulose dispersion (0.1 wt.%) made by microfluidization with 200 μm (twice) and 100 μm hole sized chambers at 1800 bar using LM20 Microfluidizer (Microfluidics, Westwood, MA, USA). Furthermore, the wet cake was made by draining the dispersion over a membrane (Durapore, 0.65 µm hydrophilic PVDF “DVPP”, Merck Millipore, Burlington, MA, USA) in a funnel, driven by vacuum. Obtained cellulose cakes were dried at 140 °C to produce pre-filters and 80 °C for filter papers using hot-press (Carver Model 4122CE, Carver, Wabash, IN, USA).

### 2.3. Dissolution of Factor IX-rich PCC

Lyophilized FIX-rich PCC samples were reconstituted by dissolving in phosphate-buffered saline (PBS). No visible particles could be seen after reconstitution, and the solution was clear and transparent. Upon dissolution, the conductivity and pH values were 15.4 mS cm^−1^ and 7.4, respectively. 

### 2.4. Filtration Setup

Pre-filtration and filtration steps were performed in a 47 mm diameter Advantech KST 47 filter holder. Prior to filtration, the pre- and filter papers were wetted in order to extrude the air by running 20 mL of PBS. The pre-filtration steps with 6 and 11 μm pre-filters were performed at 1 bar transmembrane pressure, and the filtrations with 33 μm filters were carried out at 1 or 3 bar. The permeate solutions were collected and for filtrations of the larger volume, permeate was collected in one or three fractions and saved.

### 2.5. Scanning Electron Microscopy (SEM)

For top-view images samples were fixed onto aluminum stubs with double-adhesive carbon tape, and for cross-section images the samples were mounted onto aluminum sample holders with screw. Imaging was performed using Zeiss Merlin FEG-SEM instrument (Jena, Germany). To reduce charging effects samples were sputtered with Au/Pd with a sputter coater (Polaron, Ashford, UK) was used. The sputtering settings were 4 × 10^2^ mbar and 35 mA, and the sputtering time was 30 s.

### 2.6. Cryoporometry by Differential Scanning Calorimetry

Filter paper samples (1.5–2 mg) were soaked into deionized water overnight at room temperature. Water was decanted, and the samples were placed in aluminum crucibles with a lid. Samples were cooled down to 248.15 K (−25 °C) at a rate of 10 K min^−1^ followed by heating to 277.15 K (4 °C) at a rate of 0.7 K min^−1^. Measurements were performed in five replicates.

The pore size was calculated according to Landry [41]:

(1) $$\Delta {Τ}_{\;}=-\frac{19.082}{r_{p}-1.12}-0.1207\;\;$$

where *r_p_* is the radius of pore (nm) and *ΔT* is the difference between the peak maximum for melting of pore-confined water and peak value for melting of bulk water, experimentally determined at 0.6 ± 0.01 °C.

### 2.7. Dynamic Light Scattering 

Particle size distribution was obtained from dynamic light scattering (DLS) using a Zetasizer Nano ZS (Malvern, UK) instrument. All experiments were performed in triplicates.

### 2.8. Polyacrylamide Gel Electrophoresis

Protein separation was performed by reducing polyacrylamide gel electrophoresis (SDS-PAGE). Samples were diluted (1:20 *v*/*v*) with PBS and Laemmli buffer, and boiled for 10 min. Electrophoretic separation was carried out at 270 V with Mini-PROTEAN Tetra Vertical Electrophoresis Cell (Bio-Rad, Hercules, CA, USA). Protein bands were detected with Gel Doc™ EZ System (Bio-Rad, Hercules, CA, USA), and quantified using Image Lab 6.0 analysis software (Bio-Rad).

### 2.9. Analytical SEHPLC

Samples were analyzed by size-exclusion high-pressure liquid chromatography using Hitachi Chromaster HPLC-UV system with bioZen 1.8 μm SEC-3 (Phenomenex, Torrance, CA, USA) analytical column. Chromatography was performed with 100 mM sodium phosphate, pH 6.8 mobile phase at 0.3 mL min^−1^ flow rate for 20 min. 

### 2.10. LCMS

Equal amounts (20 µg) of protein samples were taken out for digestion. After reduction and alkylation, the proteins were on-filter digested by trypsin using 3 kDa centrifugal filters (Millipore Tullagreen, Ireland) according to a standard operating procedure. Obtained peptides were dried using a speedvac system. Pellets were resolved in 60 μL of 0.1% formic acid and further diluted four times prior to nano-LCMS/MS. Tandem mass spectrometry was performed by applying HCD in the QEx-Orbitrap mass spectrometer (Thermo Finnigan, San Jose, CA, USA), equipped with a reversed-phase C18-column by 35 min long gradient. 

Database searches were performed using the Sequest algorithm, embedded in Proteome Discoverer 1.4 (Thermo Fisher Scientific, Waltham, MA, USA) against Homo Sapience proteome extracted from Uniprot, Release June 2019 with 95% confidence level per protein.

### 2.11. Bacteriophage Filtration and Titration

Coliphages PR772 and ΦX174 were spiked to the pre-filtered solutions to obtain final titer about 10^6^ plaque forming units (PFU) mL^−1^ before filtration was performed. Bacteriophage titer was determined by PFU assay by double agar overlay method. Briefly, ten-fold serially diluted bacteriophage samples were mixed with host *E. coli* strains and melted soft agar, and poured on the surface of prepared hard agar plate, followed by incubation at 37 °C for 5 h. 

Bacteriophage titer was calculated using Equation (1):

(2) $$log_{10}\left(PFU\;{\mathrm{mL}}^{-1}\right)=log_{10}\left(\frac{N\;}{V\cdot d\;}\right)$$

where *N* is the number of plaques, V is the volume (typically 0.1 mL) of added virus and d is the dilution factor.

The virus retention was expressed as *log*_10_ reduction value (*LRV*):

(3) $$LRV=log_{10}\frac{PFU_{feed}}{PFU_{permeate}}$$

## 3. Results

### 3.1. One-Step 33 μm Filtration of FIX-Rich PCC

**Figure 1:**
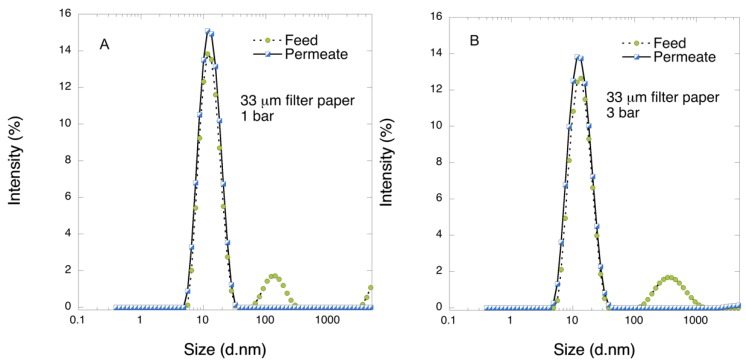
DLS profiles of feed and permeate samples after 1-step filtration through 33 μm filter paper at 1 and 3 bar.

When the FIX-rich PCC at 20 L m^−2^ volumetric load was filtered through the 33 μm mille-feuille filter paper, a rapid flux decline was observed, e.g., from about 80 L m^−2^ h^−1^ to about 10 L m^−2^ h^−1^ at 3 bar overhead pressure. DLS analysis of the feed and permeate samples revealed that the feed sample showed widely distributed fraction of protein impurities above 70 nm, which were not detectable after filtration as shown in Figure 1. Notably, these large-size impurities could not be detected in the volume distribution profiles of the feed sample but only in the intensity distribution plots, which suggests that the original amount of the aggregates is small. In the permeate sample, no particle fractions above 40 nm were detected by DLS. 

**Figure 2:**
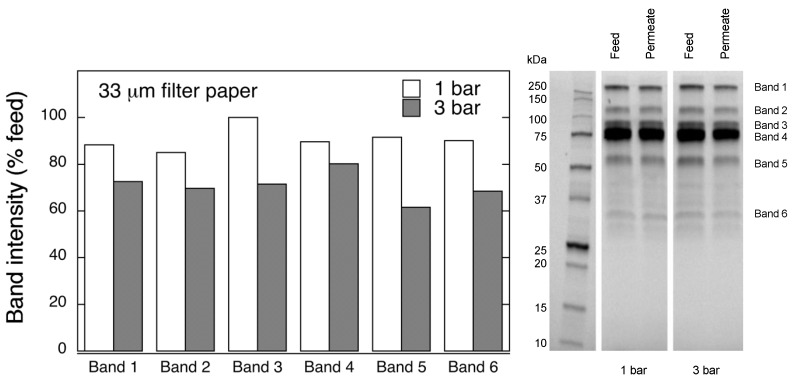
SDS-PAGE analysis of permeate fractions after 1 step filtration through 33 μm filter paper at 1 and 3 bar.

To investigate if significant changes were recorded in the protein molecular weight distribution in the permeate sample, SDS-PAGE analysis was performed, as shown in Figure 2. Additional proteomics analysis of the detected bands was not performed as it was outside of the scope of the present work. It is seen in Figure 2 that all major fractions in the permeate sample were reduced compared to the feed. The observed decrease in total protein fraction is concordant to that reported earlier for PCC product filtered through Planova 15N filter [14]. In all, it appears that the large molecular weight protein fractions are the main reason for the observed fouling. 

### 3.2. Development and Validation of Two-Step Size-Exclusion Bioprocess for FIX-Rich PCC Nanofiltration

The nanocellulose-based filter paper platform provides the possibilities to relatively easily tailor the pore-size distribution of the filter paper to a specific cut-off value. This could be achieved for instance by varying the thickness of the filter paper. 

**Figure 3:**
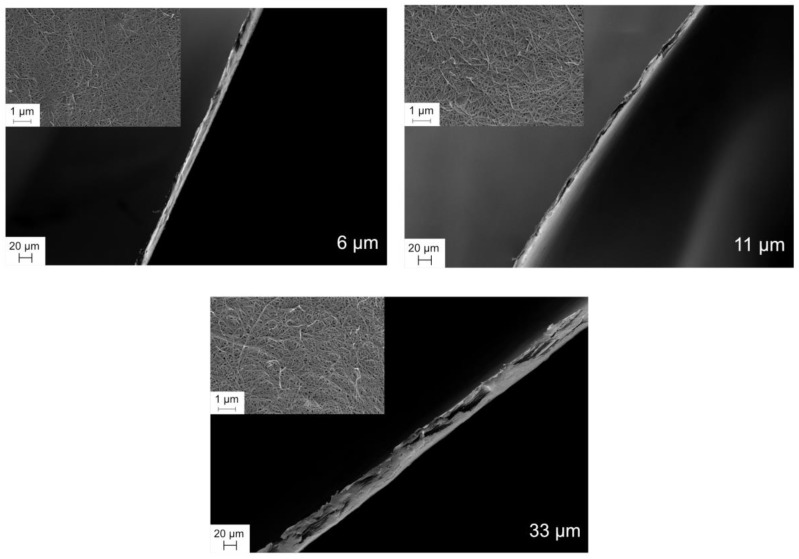
SEM images of top-view and cross-sections of 6, 11, and 33 μm filter papers.

Figure 3 shows the SEM images of the filters with varying thickness, including their top-view and cross-section. It is seen from the images of the cross-sections of the filters that they indeed feature varying thicknesses. 

**Figure 4:**
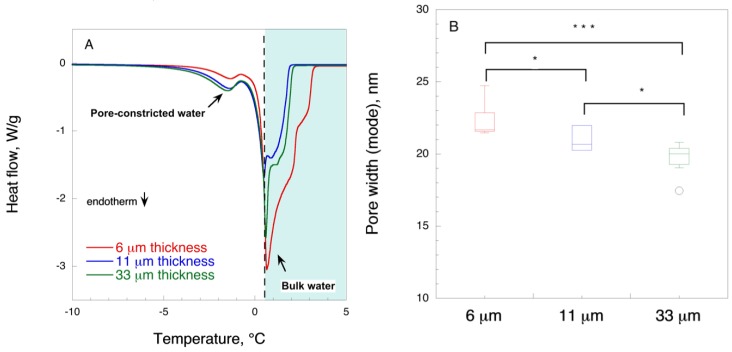
Typical cryoporometry DSC curves (**A**) and boxplot of pore width modes (**B**) for 6, 11, and 33 μm filter papers (*n* = 7). * *p* < 0.05, *** *p* < 0.01.

To derive information about the pore size of the filter cryoporometry analysis was performed. Figure 4 shows the typical CP-DSC curves of the studied samples and the boxplots of the derived pore width modes. Cryoporometry analysis has the benefit that it probes the pores in the wet state, and it is a relatively quick and highly automated and reliable method. In this method, the samples are first frozen to −40 °C and then slowly thawed. As the ice crystals start to melt, there is a detectable endotherm peak. When the water is located inside mesopores (i.e., 2–50 nm pore width), there will be a melting point depression as opposed to bulk water, present outside pores or in macropores (above 50 nm). In our experiments, bulk water melts at around 0.6 °C. The larger the melting point depression, the smaller are the pores. As seen from Figure 4 there is a trend of decreasing pore width mode with increasing thickness. 

To assess the particle rejection cut-off for each filter, model probes with 2 different particle sizes were used in the form of bacteriophages, i.e., PR772 (70 nm) and ΦX174 (28 nm) phages, see Table 1 and Table 2, respectively. These probes provide a highly sensitive tool for assessing the size-dependent rejection capability of the filters with varying thickness, i.e., 6, 11, and 33 μm. The 33 µm mille-feuille filter paper shows the lowest hydraulic flux, i.e., 38 L m^−2^ h^−1^ bar^−1^, and the highest virus removal capacity for both small- and large-size viruses, i.e., LRV ≥5.7. The 6 µm filter in the series exhibits the fastest flux, i.e., 405 L m^−2^ h^−1^ bar^−1^, but poor virus removal capacity, i.e., LRV <1 and <2 for 28 nm and 70 nm model phages. The flux and virus removal properties of 11 µm filter are intermediate to the other two filters, wherein the 11 µm filter paper shows high clearance towards 70 nm virus, i.e., >5.7, and moderate clearance toward 28 nm one, i.e., LRV 3.5–4.5, and hydraulic flux of 125 L m^−2^ h^−1^ bar^−1^. Interestingly, the small-size virus removal capacity of 11 µm filter decreased with increasing load volume, whereas that of 33 µm filter remained unaffected under the experimental conditions. The latter could probably be due to redistribution of flow through the larger pores when the smaller pores become clogged in 11 µm filter paper. 

### 3.3. Two-Step 6 μm/33 μm Filtration of FIX-Rich PCC

**Figure 5:**
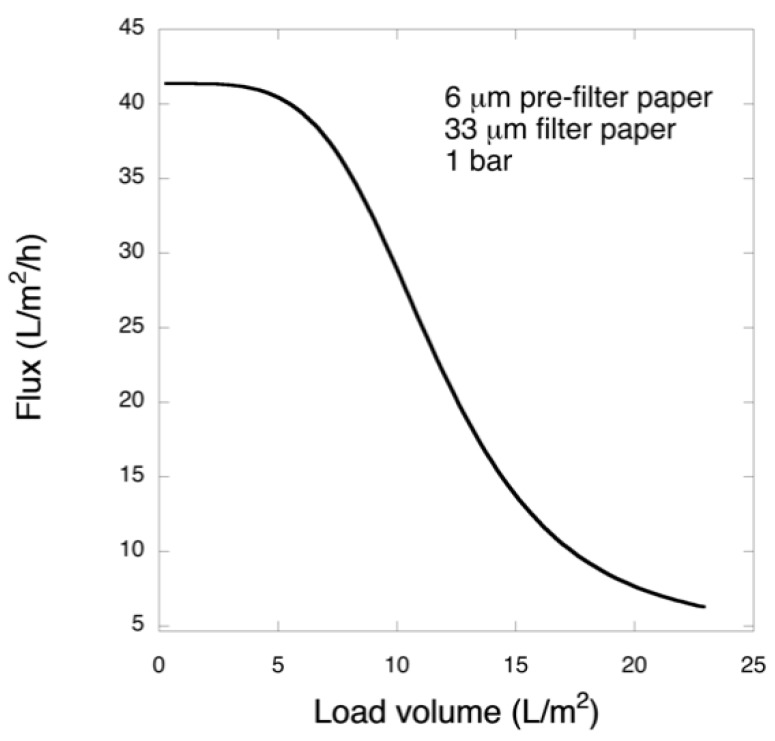
Observed permeate flux for two-step 6 μm/33 μm µm filtration at 1 bar.

Figure 5 shows the permeate flux through the 6 μm/33 μm filtration sequence at 1 bar. Rapid flux decline was observed for the permeate after initial plateau. Please note that the flux of pre-filtrate was so fast that it was not recorded.

**Figure 6:**
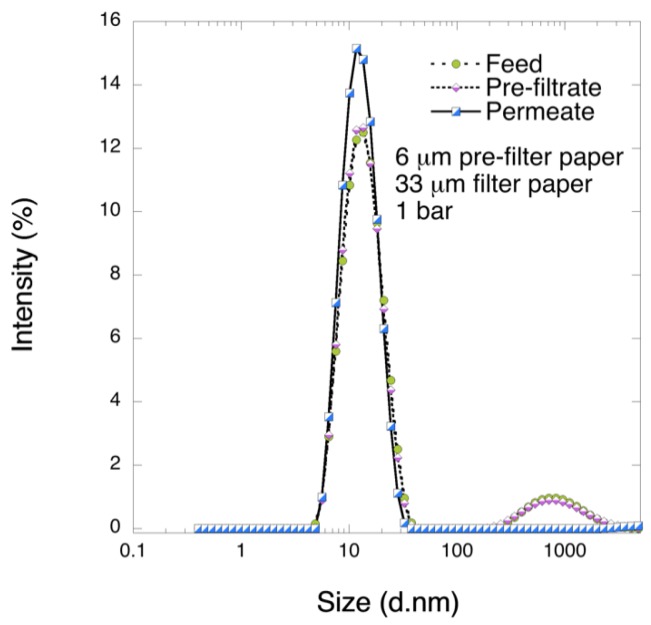
DLS analysis 11 µm filter with further filtration with 33 µm filter at 1 bar TMP DLS profiles of feed, pre-filtrate, and permeate samples after 2-step filtration through 11 µm/33 μm filter paper at 1 bar.

Figure 6 shows the DLS results for pre-filtrate and permeate samples for the 6 μm/33 μm filtration sequence at 1 bar. It was observed the fraction of large colloids was not removed by 6 μm pre-filtration. However, no aggregates were observed in the permeate sample after filtration through 33 μm filter. 

**Figure 7:**
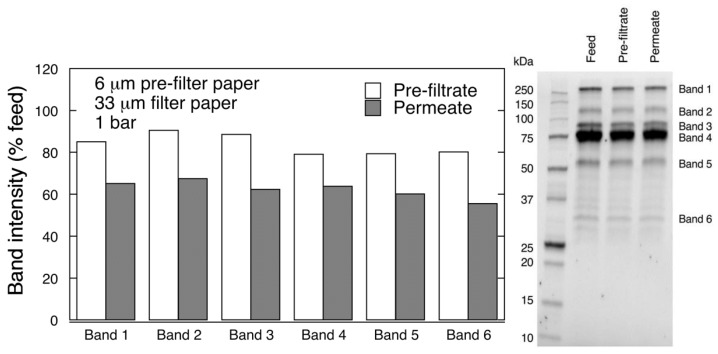
SDS-PAGE analysis of pre-filtrate and permeate fractions after 2-step filtration through 6 μm/33 μm filter paper at 1 bar.

Figure 7 shows the results of SDS-PAGE analysis of the collected samples. It is seen from the graph that all bands showed decreasing intensity. Even after 6 μm pre-filtration, some decline in the band intensity was observed. The bands for lower Mw fractions, i.e., bands 4–6, were reduced to a greater extent after pre-filtration than those of the larger Mw, i.e., bands 1–3. In the permeate sample all band intensities were further decreased. LCMS analysis suggested that key coagulation factors IX, X, V as well as prothrombin were not removed following the two-step 6 μm/33 μm filtration sequence, as shown in Appendix Table A1, Table A2 and Table A3.

Overall, the results from the filtration with 6 μm/33 μm sequence suggest that the large Mw impurities were not removed during the pre-filtration step and, subsequently, caused filter fouling and thereby low product yield during the second step. 

### 3.4. Two-Step 11 μm/33 μm Filtration of FIX-Rich PCC

**Figure 8:**
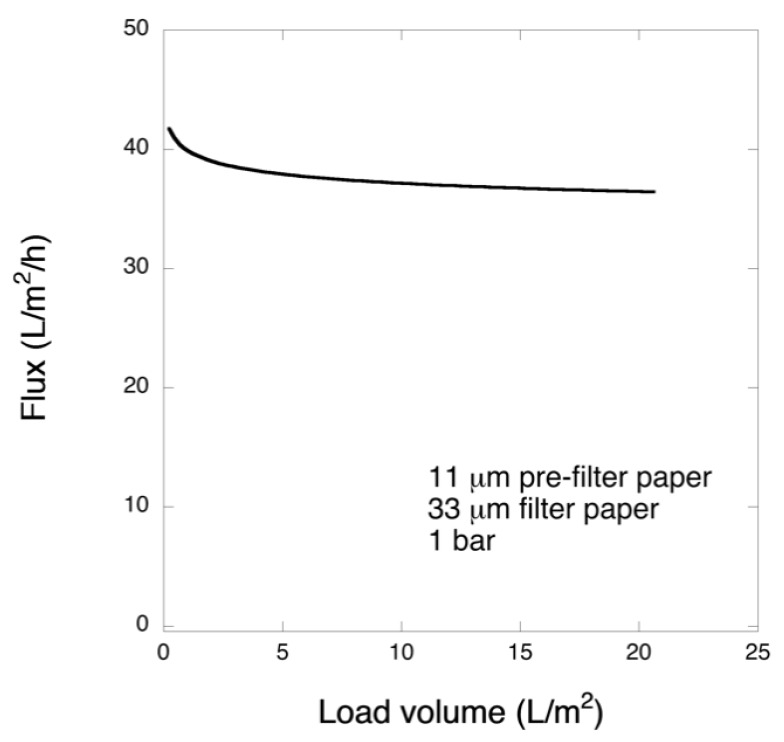
Observed permeate flux for two-step 11 μm/33 μm µm filtration at 1 bar.

In another set of experiments, the pre-filtration was performed using 11-μm filter paper followed by filtration with 33 μm filter at 1 bar. Figure 8 shows the flux data of permeate for 11 μm/33 μm filtration sequence. Increasing the thickness of the pre-filter from 6 to 11 μm significantly affected the results. The flux values for pre-filtration indicated rapid fouling as observed above for 33 μm filtration. However, in the second step of 11 μm/33 μm filtration sequence, i.e., through 33 μm filters, stable flux was observed for the entire processed volume, Figure 7. The results contrast starkly those observed for 6 μm/33 μm filtration sequence as shown in Figure 4.

**Figure 9:**
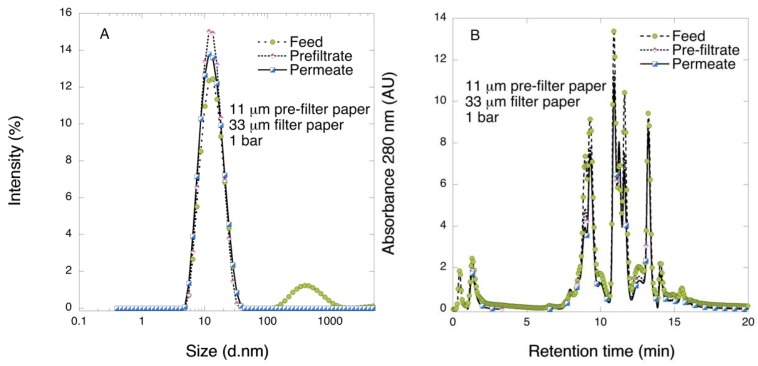
DLS (**A**) and SEHPLC (**B**) profiles of feed, pre-filtrate, and permeate samples after two-step filtration through 11 µm/33 μm filter paper at 1 bar.

Figure 9 shows the results of DLS analysis of the pre-filtrate and permeate samples. It is seen that the fraction of large-size impurities, which was clearly visible in the feed solution, was absent both in the pre-filtrate and permeate fractions of 11 μm/33 μm filtration sequence. The latter suggests that pre-filtration with 11 μm filter paper efficiently removes the large-size impurities, unlike pre-filtration with 6 μm filter paper. Additional SEHPLC analysis was performed on these samples as shown in Figure 8. It is seen in the graph that the peak retention times and relative intensities are similar in all three samples except for the early peak at 0.5 min in the feed sample. This peak, which corresponds to the largest protein fraction was not detectable in pre-filtrate and permeate samples. 

**Figure 10:**
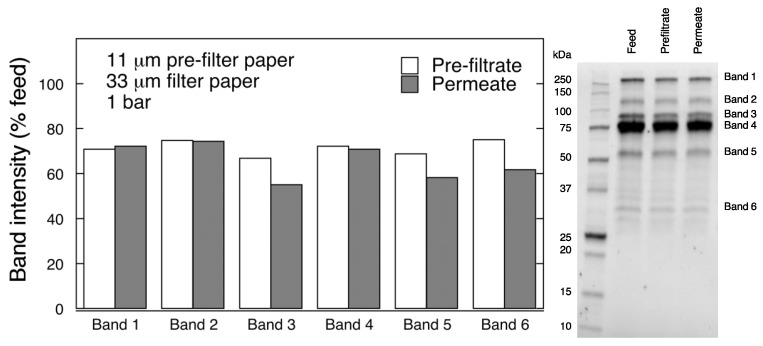
SDS-PAGE analysis of pre-filtrate and permeate fractions after 2-step filtration through 11 μm/33 μm filter paper at 1 bar.

The results of the SDS-PAGE analysis for 11 μm /33 μm filtration sequence are summarized in Figure 10. It is seen from the graph that the band intensities were reduced in the pre-filtrate and permeate samples as compared to the feed. It should be noted that in general the band intensities were reduced to a greater extent after pre-filtration with 11 μm filter than with 6 μm filter. The decrease of band intensity levels in the permeate sample passed through the 33 μm filter after 11 μm filtration was much less drastic than that for 6 μm/33 μm filtration sequence. In particular, no significant changes were observed for bands 1, 2, and 4. For bands 3, 5, and 6 some intensity reduction was further detected in the permeate sample. LCMS analysis suggested that key coagulation factors IX, X, V as well as prothrombin were not removed following the two-step 11 μm/33 μm filtration sequence (for details see Appendix Table A1, Table A4 and Table A5).

**Figure 11:**
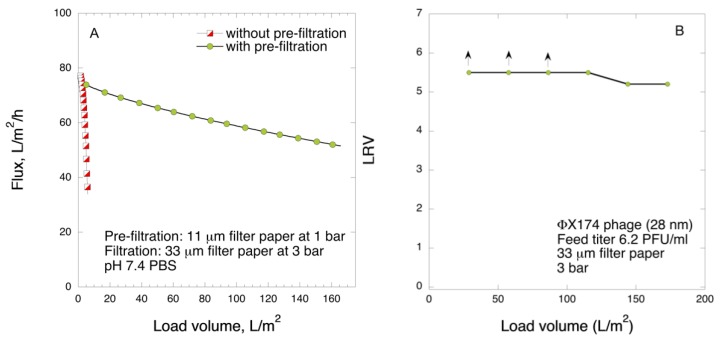
Observed flux for FIX-rich PCC permeate for 2-step 11 μm/33 μm filtration with 175 L m^−2^ load volume at 3 bar (**A**) and LRV for ΦX174 phage (**B**).

Based on the above results, it was concluded that pre-filtration with 11 µm pre-filter removes the aggregates, which in turn greatly enhances the yield of the 33 µm filtration. To confirm the high virus removal capacity of 33 µm filter, the filter paper was loaded with much larger volume than that tested earlier, i.e., 175 L m^−2^. Figure 11 shows the result of the large load filtration. Following the filtration, no abrupt filter fouling was detected for the entire processed volume, although some flux decline could be observed (Figure 11A). Under the experimental conditions, it is estimated that Vmax of the process will be roughly around 500 L m^−2^, which is a drastic improvement from 20 L m^−2^ when filtering in a single-step process through 33 µm filter paper. Furthermore, the filter paper showed high model small-size virus removal capacity, wherein LRV was ≥ 5 in all collected fractions (Figure 11B). In particular, no detectable PFUs were observed at all up to 90 L m^−2^ load volume. In the last fractions only residual breakthrough (1–2 PFUs per agar plate, corresponding to 0.7 PFU mL^−1^) was detected. Thus, it was confirmed that the two-step 11 µm/33 µm filtration provides enhanced throughput and good capacity to remove small-size virus without abrupt fouling even when challenged with a relatively large load. 

## 4. Discussion

In this article, the filtration of a highly challenging hematologic product was investigated. Considering that FIX-rich PCC inherently consists of many bioactive components and some impurities, the virus removal filtration of this product is difficult without fouling. The virus removal filtration of PCC was previously reported using Ultipor DV50 filters, which are dedicated for removal of large-size viruses but do not ensure viral safety against parvoviruses [2]. Filtration of PCC through small-size virus removal filters, e.g., Planova 15N, resulted in nearly 39% total protein loss and reduced FIX and FII activity, which was ascribed to presence of large-size complexes between clotting factors and high molecular weight impurities [14]. It was further reported in the same study that filtration of highly purified FIX through Planova 15N not only did not result in the decrease of FIX activity but also improved its purity [14].

**Figure 12:**
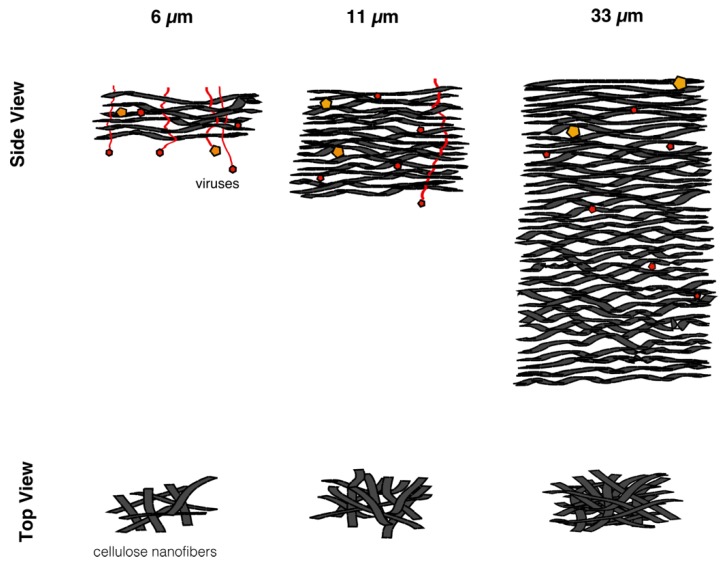
Illustration of the mechanism of virus removal with increased thickness of nanocellulose-based filter paper. Yellow symbols represent large-size model virus and red symbols represent small-size viruses. Increased thickness of the filter results in tighter pores and enhanced virus clearance.

In this work, in order to achieve high virus removal capacity combined with reduced fouling, a tailored two-step process of filtration with nanocellulose-based filter paper was developed. In particular, sacrificial pre-filters with a thickness of 6 and 11 μm were tested. The increased thickness of the filters resulted in tighter pore structure as detected by cryoporometry. The observed effect is explained as follows and illustrated in Figure 12. The mille-feuille filter paper consists of a stratified 3-dimensional network of cellulose nanofibers, producing a mesh-like stricture. The layered structure is illustrated in the side-view panel of Figure 12. Considering that the nanofibers are randomly distributed in each layer, the pores, which percolate throughout the entire depth of the filter, become tighter with increasing number of layers. The latter is reflected, e.g., in improved virus clearance properties with increased thickness or enhanced aggregate removal properties. Based on the results of PFU titrations of 27 and 70 nm phage particles, it was concluded that the tested filters show varying particle size rejection threshold as the thickness of the filter is increased. Thus, the observed effect is due to the combination of the receding pore size and depth effects (increased tortuosity). The latter enables using pre-filters with tailored cut-off to remove protein aggregates, which eventually results in improved flux through the dedicated virus removal filter. 

Overall, the two-step approach presented here is based on the size-exclusion principles and is therefore robust. It could thus further be adapted in the manufacturing of other protein-based pharmaceutics, too, including recombinant proteins wherein impurities in the form of host cell proteins may greatly affect the final yield of the biologics during virus removal nanofiltration.

## 5. Conclusions

A two-step process was developed to both enhance filtration capacity (25-fold) and achieve high clearance of small-size viruses (LRV >5) using appropriate pre-filter paper. Large-size aggregates were the main foulants in the feed solution, and by tailoring the properties of the pre-filters the foulants were efficiently removed. In particular, 11 μm/33 μm filtration was found most suitable. The presented approach could potentially be applied for bioprocessing other protein-based drugs, both derived from plasma and produced by recombinant approaches. The article further provides new insights regarding the mechanism of virus removal in the nanocellulose-based filter paper, highlighting the combined effect of size exclusion and tortuosity of pore network.

## Figures and Tables

**Table 1:** LRVs for 70 nm (PR772) bacteriophages filtered through 6, 11, and 33 μm filter papers. The results represent the virus clearance data of virus-spiked PBS. Green color code denotes high virus clearance LRV > 5; Yellow denotes moderate virus clearance (2 < LRV < 5); and pink denotes low virus clearance (LRV < 1).

Thickness (μm)	Load Volume		
	7.5 L m^−2^	15 L m^−2^	23 L m^−2^
6	1.6 ± 0.2	1.3 ± 0.1	1.1 ± 0.3
11	>5.5 ± 0.2	>5.5 ± 0.2	>5.5 ± 0.2
33	>5.7 ± 0.2	>5.7 ± 0.2	>5.7 ± 0.2

**Table 2:** LRVs for 28 nm (ΦΧ174) bacteriophages filtered through 6, 11, and 33 μm filter papers. The results represent the virus clearance data of virus-spiked PBS. Green color code denotes high virus clearance LRV > 5; Yellow denotes moderate virus clearance (2 < LRV < 5); and pink denotes low virus clearance (LRV < 2).

Thickness (μm)	Load Volume		
	7.5 L m^−2^	15 L m^−2^	23 L m^−2^
6	0.7 ± 0.3	0.8 ± 0.3	0.8 ± 0.2
11	4.5 ± 0.5	3.7 ± 0.4	3.5 ± 0.7
33	>5.7 ± 0.4	>5.7 ± 0.4	5.7 ± 0.4

## Appendix A

**Table A1:** LCMS analysis of FIX-PCC feed solution.

Accession	Description	Score	Coverage	MW [kDa]	calc. pI
P0C0L5	Complement C4-B	1920.89	76.38	192.6	7.27
P0C0L4	Complement C4-A	1898.90	76.03	192.7	7.08
P00734	Prothrombin	1767.89	75.56	70.0	5.90
P19823	Inter-alpha-trypsin inhibitor heavy chain H2	917.12	53.59	106.4	6.86
P19827	Inter-alpha-trypsin inhibitor heavy chain H1	685.72	55.76	101.3	6.79
Q06033	Inter-alpha-trypsin inhibitor heavy chain H3	257.18	40.22	99.8	5.74
P02760	Protein AMBP	199.11	27.56	39.0	6.25
P00740	Coagulation factor IX	134.15	47.29	51.7	5.47
P00742	Coagulation factor X	59.86	38.52	54.7	5.94
P02768	Serum albumin	48.49	28.41	69.3	6.28
P49747	Cartilage oligomeric matrix protein	41.93	14.27	82.8	4.60
P67936	Tropomyosin alpha-4 chain	32.67	39.11	28.5	4.69
P01857	Immunoglobulin heavy constant gamma 1	32.57	35.15	36.1	8.19
P01834	Immunoglobulin kappa constant	30.33	39.25	11.8	6.52
P07225	Vitamin K-dependent protein S	26.15	12.72	75.1	5.67
P0DOY3	Immunoglobulin lambda constant 3	21.63	78.30	11.3	7.24
P51884	Lumican	21.32	19.53	38.4	6.61
B9A064	Immunoglobulin lambda-like polypeptide 5	19.49	41.12	23.0	8.84
P04004	Vitronectin	19.13	14.02	54.3	5.80
P01861	Immunoglobulin heavy constant gamma 4	19.09	20.18	35.9	7.36
Q08380	Galectin-3-binding protein	18.16	9.74	65.3	5.27
P07359	Platelet glycoprotein Ib alpha chain	17.06	8.28	71.5	6.29
P35443	Thrombospondin-4	16.88	6.66	105.8	4.68
P01876	Immunoglobulin heavy constant alpha 1	15.74	24.93	37.6	6.51
P01859	Immunoglobulin heavy constant gamma 2	13.53	21.78	35.9	7.59
P01871	Immunoglobulin heavy constant mu	8.22	4.64	49.4	6.77
Q14515	SPARC-like protein 1	7.09	6.93	75.2	4.81
P04070	Vitamin K-dependent protein C	5.36	7.16	52.0	6.28
O95810	Caveolae-associated protein 2	4.07	4.71	47.1	5.21
P16070	CD44 antigen	3.37	2.83	81.5	5.33
Q99436	Proteasome subunit beta type-7	2.23	8.30	29.9	7.68
P12259	Coagulation factor V	1.95	0.81	251.5	6.05
P07237	Protein disulfide-isomerase	1.69	3.35	57.1	4.87
P10909	Clusterin	1.53	4.01	52.5	6.27

**Table A2:** LCMS analysis of FIX-PCC pre-filtrate following filtration with 6 μm filter.

Accession	Description	Score	Coverage	MW [kDa]	calc. pI
P00734	Prothrombin	2584.88	71.86	70.0	5.90
P0C0L5	Complement C4-B	2441.20	77.69	192.6	7.27
P0C0L4	Complement C4-A	2393.34	74.89	192.7	7.08
P19827	Inter-alpha-trypsin inhibitor heavy chain H1	1154.64	54.34	101.3	6.79
P19823	Inter-alpha-trypsin inhibitor heavy chain H2	1136.27	55.50	106.4	6.86
P02760	Protein AMBP	337.33	25.57	39.0	6.25
Q06033	Inter-alpha-trypsin inhibitor heavy chain H3	332.27	43.15	99.8	5.74
P00740	Coagulation factor IX	151.88	54.66	51.7	5.47
P00742	Coagulation factor X	81.34	41.19	54.7	5.94
P02768	Serum albumin	60.16	37.44	69.3	6.28
P01834	Immunoglobulin kappa constant OS=Homo sapiens	48.66	81.31	11.8	6.52
P51884	Lumican	42.56	35.50	38.4	6.61
P49747	Cartilage oligomeric matrix protein	41.14	16.12	82.8	4.60
P01857	Immunoglobulin heavy constant gamma 1	40.75	41.21	36.1	8.19
P67936	Tropomyosin alpha-4 chain	31.98	36.29	28.5	4.69
P01861	Immunoglobulin heavy constant gamma 4	30.25	28.13	35.9	7.36
P01860	Immunoglobulin heavy constant gamma 3	28.46	27.06	41.3	7.90
P04004	Vitronectin OS=Homo sapiens	25.83	17.99	54.3	5.80
P0DOY2	Immunoglobulin lambda constant 2	23.29	67.92	11.3	7.24
P07225	Vitamin K-dependent protein S	21.00	11.83	75.1	5.67
P07359	Platelet glycoprotein Ib alpha chain	20.03	8.28	71.5	6.29
Q08380	Galectin-3-binding protein	17.21	12.82	65.3	5.27
P01876	Immunoglobulin heavy constant alpha 1	15.47	19.55	37.6	6.51
P35443	Thrombospondin-4	14.04	4.79	105.8	4.68
P01859	Immunoglobulin heavy constant gamma 2	12.71	27.91	35.9	7.59
P01024	Complement C3	7.05	1.56	187.0	6.40
P12259	Coagulation factor V	6.08	1.57	251.5	6.05
Q14515	SPARC-like protein 1	5.71	6.93	75.2	4.81
P16070	CD44 antigen	5.36	2.70	81.5	5.33
P13591	Neural cell adhesion molecule 1	5.24	2.56	94.5	4.87
P04070	Vitamin K-dependent protein C	5.14	5.86	52.0	6.28
P07900	Heat shock protein HSP 90-alpha	4.64	4.92	84.6	5.02
P61981	14-3-3 protein gamma	3.79	7.29	28.3	4.89
P63104	14-3-3 protein zeta/delta	3.48	6.94	27.7	4.79
Q99436	Proteasome subunit beta type-7	2.11	8.30	29.9	7.68
P25786	Proteasome subunit alpha type-1	1.66	7.98	29.5	6.61

**Table A3:** LCMS analysis of FIX-PCC permeate following two-step filtration with 6 μm/33 μm filters.

Accession	Description	Score	Coverage	MW [kDa]	calc. pI
P00734	Prothrombin	2759.75	73.31	70.0	5.90
P0C0L5	Complement C4-B	2411.85	78.61	192.6	7.27
P0C0L4	Complement C4-A	2379.43	75.80	192.7	7.08
P19823	Inter-alpha-trypsin inhibitor heavy chain H2	1480.56	56.77	106.4	6.86
P19827	Inter-alpha-trypsin inhibitor heavy chain H1	1167.61	54.45	101.3	6.79
Q06033	Inter-alpha-trypsin inhibitor heavy chain H3	352.03	43.60	99.8	5.74
P02760	Protein AMBP	263.80	27.56	39.0	6.25
P00740	Coagulation factor IX	216.46	54.66	51.7	5.47
P00742	Coagulation factor X	90.46	37.09	54.7	5.94
P02768	Serum albumin	62.34	36.45	69.3	6.28
P01857	Immunoglobulin heavy constant gamma 1	47.83	39.09	36.1	8.19
P04004	Vitronectin	45.60	23.64	54.3	5.80
P49747	Cartilage oligomeric matrix protein	43.78	19.82	82.8	4.60
P51884	Lumican OS=Homo sapiens	43.31	42.90	38.4	6.61
P01834	Immunoglobulin kappa constant	40.16	49.53	11.8	6.52
P67936	Tropomyosin alpha-4 chain	39.83	40.32	28.5	4.69
P07359	Platelet glycoprotein Ib alpha chain	31.44	12.12	71.5	6.29
P0DOY2	Immunoglobulin lambda constant 2	29.10	75.47	11.3	7.24
Q08380	Galectin-3-binding protein	28.60	18.80	65.3	5.27
P01861	Immunoglobulin heavy constant gamma 4	28.41	22.02	35.9	7.36
P01876	Immunoglobulin heavy constant alpha 1	23.24	25.78	37.6	6.51
B9A064	Immunoglobulin lambda-like polypeptide 5	22.01	38.79	23.0	8.84
P07225	Vitamin K-dependent protein S	20.55	14.35	75.1	5.67
P01859	Immunoglobulin heavy constant gamma 2	19.80	24.23	35.9	7.59
Q14515	SPARC-like protein 1	19.72	9.19	75.2	4.81
P35443	Thrombospondin-4	14.73	4.79	105.8	4.68
P12259	Coagulation factor V	8.97	2.34	251.5	6.05
P13591	Neural cell adhesion molecule 1	8.03	4.55	94.5	4.87
P12814	Alpha-actinin-1	7.56	3.48	103.0	5.41
P0CG38	POTE ankyrin domain family member I	7.35	3.07	121.2	6.21
P01871	Immunoglobulin heavy constant mu	7.06	8.61	49.4	6.77
P04070	Vitamin K-dependent protein C	5.93	10.63	52.0	6.28
P16070	CD44 antigen	5.80	2.70	81.5	5.33
P61981	14-3-3 protein gamma	5.53	10.93	28.3	4.89
P07900	Heat shock protein HSP 90-alpha	3.08	3.42	84.6	5.02
P01023	Alpha-2-macroglobulin	2.56	1.76	163.2	6.46
Q14185	Dedicator of cytokinesis protein 1	1.99	1.66	215.2	7.56
Q562R1	Beta-actin-like protein 2	1.78	7.71	42.0	5.59

**Table A4:** LCMS analysis of FIX-PCC pre-filtrate following filtration with 11 μm filter.

Accession	Description	Score	Coverage	MW [kDa]	calc. pI
P00734	Prothrombin	2458.17	67.20	70.0	5.90
P0C0L5	Complement C4-B	2389.47	80.68	192.6	7.27
P0C0L4	Complement C4-A	2352.05	79.59	192.7	7.08
P19823	Inter-alpha-trypsin inhibitor heavy chain H2	1268.03	56.87	106.4	6.86
P19827	Inter-alpha-trypsin inhibitor heavy chain H1	1122.57	59.50	101.3	6.79
Q06033	Inter-alpha-trypsin inhibitor heavy chain H3	352.56	40.00	99.8	5.74
P02760	Protein AMBP	285.56	27.56	39.0	6.25
P00740	Coagulation factor IX	182.33	57.27	51.7	5.47
P00742	Coagulation factor X	80.33	41.19	54.7	5.94
P01834	Immunoglobulin kappa constant	54.45	54.21	11.8	6.52
P49747	Cartilage oligomeric matrix protein	53.77	24.31	82.8	4.60
P01857	Immunoglobulin heavy constant gamma 1	45.54	43.33	36.1	8.19
P02768	Serum albumin	44.26	25.78	69.3	6.28
P07359	Platelet glycoprotein Ib alpha chain	33.83	16.56	71.5	6.29
P01860	Immunoglobulin heavy constant gamma 3	33.53	31.83	41.3	7.90
P0DOY3	Immunoglobulin lambda constant 3	33.11	70.75	11.3	7.24
P01861	Immunoglobulin heavy constant gamma 4	30.81	23.85	35.9	7.36
P04004	Vitronectin	27.21	23.64	54.3	5.80
P51884	Lumican	26.62	35.50	38.4	6.61
P01876	Immunoglobulin heavy constant alpha 1	25.67	29.18	37.6	6.51
Q08380	Galectin-3-binding protein	25.15	17.78	65.3	5.27
P67936	Tropomyosin alpha-4 chain	25.00	44.35	28.5	4.69
P01859	Immunoglobulin heavy constant gamma 2	17.12	25.77	35.9	7.59
P07225	Vitamin K-dependent protein S	16.75	9.62	75.1	5.67
P35443	Thrombospondin-4	16.66	5.52	105.8	4.68
P12259	Coagulation factor V	10.18	1.98	251.5	6.05
P04070	Vitamin K-dependent protein C	7.36	5.86	52.0	6.28
P16070	CD44 antigen	5.64	2.70	81.5	5.33
P13591	Neural cell adhesion molecule 1	5.50	1.75	94.5	4.87
P22105	Tenascin-X	4.42	2.33	458.1	5.17
Q99436	Proteasome subunit beta type-7	4.03	8.30	29.9	7.68
P12814	Alpha-actinin-1	3.68	2.35	103.0	5.41
P27348	14-3-3 protein theta	3.53	6.53	27.7	4.78
P07900	Heat shock protein HSP 90-alpha	1.73	3.14	84.6	5.02

**Table A5:** LCMS analysis of FIX-PCC permeate following two-step filtration with 11 μm/33 μm filters.

Accession	Description	Score	Coverage	MW [kDa]	calc. pI
P0C0L5	Complement C4-B	2266.18	76.26	192.6	7.27
P0C0L4	Complement C4-A	2219.29	76.26	192.7	7.08
P00734	Prothrombin	2011.89	71.54	70.0	5.90
P19823	Inter-alpha-trypsin inhibitor heavy chain H2	1452.50	55.81	106.4	6.86
P19827	Inter-alpha-trypsin inhibitor heavy chain H1	1205.49	54.23	101.3	6.79
Q06033	Inter-alpha-trypsin inhibitor heavy chain H3	256.28	46.97	99.8	5.74
P02760	Protein AMBP	213.99	25.57	39.0	6.25
P00740	Coagulation factor IX	114.46	45.55	51.7	5.47
P00742	Coagulation factor X	74.31	36.07	54.7	5.94
P02768	Serum albumin	46.51	27.91	69.3	6.28
P01857	Immunoglobulin heavy constant gamma 1	45.77	43.33	36.1	8.19
P49747	Cartilage oligomeric matrix protein	34.16	20.08	82.8	4.60
P01834	Immunoglobulin kappa constant	32.23	49.53	11.8	6.52
P67936	Tropomyosin alpha-4 chain	27.70	37.90	28.5	4.69
P01860	Immunoglobulin heavy constant gamma 3	27.37	25.99	41.3	7.90
P51884	Lumican	25.59	39.94	38.4	6.61
P01861	Immunoglobulin heavy constant gamma 4	24.73	26.61	35.9	7.36
P01859	Immunoglobulin heavy constant gamma 2	23.14	32.82	35.9	7.59
P04004	Vitronectin	22.83	23.43	54.3	5.80
P07359	Platelet glycoprotein Ib alpha chain	21.45	9.82	71.5	6.29
P07225	Vitamin K-dependent protein S	20.38	10.65	75.1	5.67
P0DOY2	Immunoglobulin lambda constant 2	17.99	37.74	11.3	7.24
Q08380	Galectin-3-binding protein	17.44	15.73	65.3	5.27
P01876	Immunoglobulin heavy constant alpha 1	17.06	25.78	37.6	6.51
P35443	Thrombospondin-4	9.76	4.79	105.8	4.68
P12259	Coagulation factor V	8.36	2.11	251.5	6.05
P04070	Vitamin K-dependent protein C	7.60	8.89	52.0	6.28
P61981	14-3-3 protein gamma	5.93	10.53	28.3	4.89
P16070	CD44 antigen	4.96	2.70	81.5	5.33
Q99436	Proteasome subunit beta type-7	4.05	8.30	29.9	7.68
Q14515	SPARC-like protein 1	4.05	3.77	75.2	4.81
P07900	Heat shock protein HSP 90-alpha	2.46	2.60	84.6	5.02
P01023	Alpha-2-macroglobulin	1.97	1.49	163.2	6.46
P22105	Tenascin-X OS=Homo sapiens	1.66	0.66	458.1	5.17
P13591	Neural cell adhesion molecule 1	0.00	1.75	94.5	4.87
Q5UIP0	Telomere-associated protein RIF1]	0.00	1.62	274.3	5.52
